# A probabilistic census-travel model to predict introduction sites of exotic plant, animal and human pathogens

**DOI:** 10.1098/rstb.2018.0260

**Published:** 2019-05-20

**Authors:** Tim Gottwald, Weiqi Luo, Drew Posny, Tim Riley, Frank Louws

**Affiliations:** 1 US Department of Agriculture, Agricultural Research Service, Fort Pierce, FL 34945, USA; 2 Center for Integrated Pest Management, North Carolina State University, Raleigh, NC 27695, USA; 3 US Department of Agriculture, Animal and Plant Health Inspection Service, Orlando, FL 32824, USA

**Keywords:** census-travel model, introductions, exotic, contagion

## Abstract

International travel offers an extensive network for new and recurring human-mediated introductions of exotic infectious pathogens and biota, freeing geographical constraints. We present a predictive census-travel model that integrates international travel with endpoint census data and epidemiological characteristics to predict points of introduction. Population demographics, inbound and outbound travel patterns, and quantification of source strength by country are combined to estimate and rank risk of introduction at user-scalable land parcel areas (e.g. state, county, zip code, census tract, gridded landscapes (1 mi^2^, 5 km^2^, etc.)). This risk ranking by parcel can be used to develop pathogen surveillance programmes, and has been incorporated in multiple US state/federal surveillance protocols. The census-travel model is versatile and independent of pathosystems, and applies a risk algorithm to generate risk maps for plant, human and animal contagions at different spatial scales. An interactive, user-friendly interface is available online (https://epi-models.shinyapps.io/Census_Travel/) to provide ease-of-use for regulatory agencies for early detection of high-risk exotics. The interface allows users to parametrize and run the model without knowledge of background code and underpinning data.

This article is part of the theme issue ‘Modelling infectious disease outbreaks in humans, animals and plants: epidemic forecasting and control’. This theme issue is linked with the earlier issue ‘Modelling infectious disease outbreaks in humans, animals and plants: approaches and important themes’.

## 1. Introduction

Emerging pests, pathogens and infectious diseases pose significant threats to public health security, agricultural productivity and ecological diversity. Globalization continually exacerbates long-distance human-mediated pathways for invasive zoonotic and botanical pathogen introduction around the world at unprecedented rates [1,2]. The most important pathway accelerating new pathogen and pest introductions is human travel, migration and trade [3–12]. Once introduced, the exotic organism begins to invade the susceptible population if suitable climatic and environmental conditions are met. Recently, awareness of the damaging impacts from non-indigenous pest and disease introduction has increased substantially, and regulatory agencies have strengthened their efforts to prevent such introductions through quarantines and other proactive protocols [8]. Reliable estimates for invasion pathways are therefore of critical importance to develop appropriate management strategies and regulatory policies. These initial introductions of exotic pathogens and pests by definition occur in very low incidence, and thus, are challenging to detect (‘finding a needle in a haystack’). Introductions can occur in animal populations dispersed across broad areas, across the entire extent of human population or across wide-ranging geographical landscapes of mixed agricultural/residential areas, from low-density rural to high-density urban areas. Finding point introductions requires substantial dedication of manpower and fiscal resources, often going undetected for prolonged time periods until incidence exceeds the lower threshold of sampling sensitivity. Only when an introduced pathogen surpasses the perception/detection threshold, do we become aware of its existence in the previously pathogen-free area and begin to react to it via attempts at control, mitigation or eradication. Yet optimizing the probability of epidemic eradication depends on early detection prior to spread. Thus, the earlier the detection, the more probable the pathogen can be eliminated or the propagation slowed, lessening the epidemic impact over multiple years [13].

Although there is a greater understanding of the importance and urgency for early detection of non-indigenous pathogens, many approaches present broad patterns of historical pathogen emergence and establishment [8,9,12], or predict post-introduction disease epidemics [14]. These are reactive, occurring after pathogen introduction, and are generalized to relatively large spatial scales, leading to inefficient manpower and resource allocation. Very few current models address initial introductions adequately, especially at sufficiently fine spatial scales, to pinpoint risk and target surveillance efforts for early detection [15,16]. Therefore, we aim to develop a risk-based census-travel model to estimate introductions of exotic pathogens and pests by exploiting international travel patterns and connectivity among human populations as a proxy for potential pathways of introduction. The model traces human-assisted pathways from source countries to elucidate high-risk areas for introduction. Specifically, we: (i) construct a mathematical model to predict points of introduction of invasive pathogens and quantify the relative risk; (ii) demonstrate its application on several case studies as well as its utility for readily integrating with other survey models and tools to develop comprehensive, proactive survey programmes; and (iii) provide a user-friendly, interactive tool to assist plant, animal and human health regulatory authorities with detecting new introductions as early as possible. The model can serve as a tool to facilitate more efficient survey design, resulting in earlier detection, and thus limiting the adverse impact of introduction.

## 2. Methods

Our motivation for this study is to predict introductions based on human travel and population connectivity for preemptive preventative action rather than rely on a delayed reaction to post-introduction and subsequent spread. To get around the ‘finding a needle in a haystack’ problem (i.e. very low prevalence of initial introductions), we present a model framework that employs a risk-based algorithm to explore pathways of potential risk introduction for geographical area(s) of interest. For a given pest or pathogen, international occurrence/prevalence data are gathered from the published literature and/or expert elicitation to ascertain the group of potential source countries, ranking their relative importance. International travel records from the collection of source countries are linked with census demographic data to estimate their endpoint destination for potential pathogen introduction. The endpoint can be explored at various spatial hierarchies (e.g. country, province, state, county, census tract, 1 mi^2^ grid, etc.) to assist survey methodologies at different scales. We demonstrate the composition and utility of the model to predict initial introductions using examples from phytobacterial, phytoviral, phytomycological and zoonotic viral vectored and non-vectored pathogens (electronic supplementary material, table S1).

For simplicity, we refer to the introduction of ‘pathogens or disease’ for the remainder of this paper with the reader's understanding that the model can be applied to a wide array of additional exotic biota types.

For model development, we use the USA as the endpoint country due to the prevalence and granularity of travel, census and demographic data available. This methodology can be applied to other endpoint countries, if similar extensive databases exist. The model parses the risk of introduction across user-scalable, spatially distinct areas (parcels), within which the susceptible human, plant or animal populations are geographically dispersed. Those parcels can be then prioritized by potential risk of introduction for the purpose of detection survey.

The model relies on three main data-driven components (electronic supplementary material, table S2).

### (a) International travel: pattern and volume

**Figure 1.**
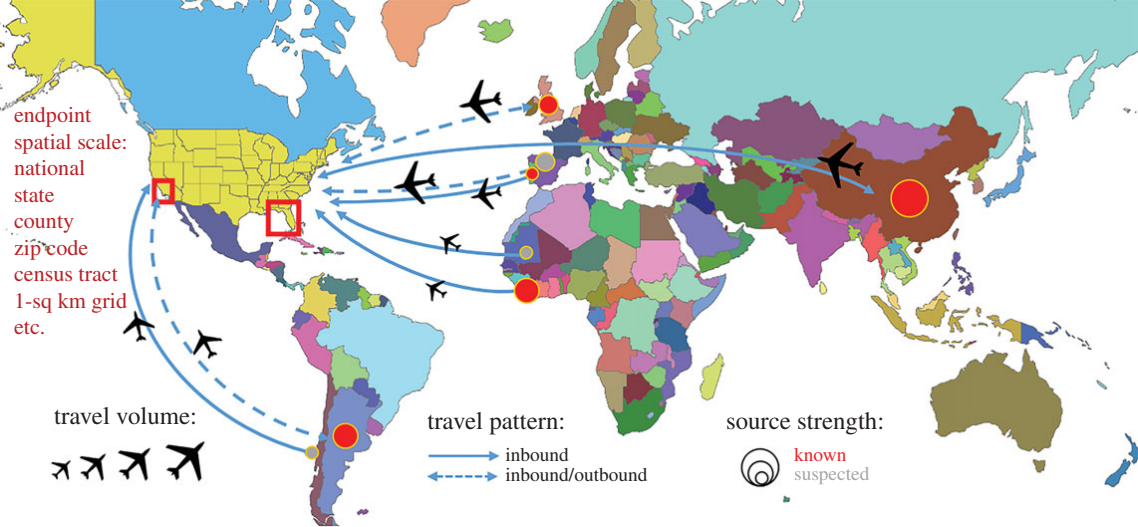
Schematic diagram to represent the conceptual pathway model for international disease spread from 176 potential pathogen-originating countries. The connectivity between known source countries, suspected neighbouring countries and the potential traveller endpoints with consideration of source strength, travel volume and endpoint demographics represents a simplification of the real global travel network structure. The census-travel model integrates all of these components to estimate risk of introduction.

The impact of international travel on the introduction and spread of infectious diseases has led to considerable concern [17]. Both international visitors and US outbound travellers can be exposed to many bacterial, viral, parasitic and fungal infections while abroad, creating various pathways of introduction [18]. Travellers can also transport infected plant and animal materials and pests illicitly or inadvertently, unaware of quarantines and import/export regulations. According to the National Travel and Tourism Office (NTTO, Department of Commerce), which publishes inbound and outbound travel data for the USA, the USA received 75.9 million international visitors in 2016. Canada, Mexico, UK, Japan and China were the five countries with the highest volume of travellers to the USA, respectively. Concurrently, US citizens travelling overseas has risen to 38.3 million in 2017, compared with 26.8 million in 2000. These historically increasing travel trends to and from the USA are predicted to continue to rise, amassing even greater opportunities for pathogen introduction. Fortuitously, the US Department of Homeland Security (DHS) captures travel volume for countries visited by outbound US residents through the Advanced Passenger Information System (formerly DHS I-92) and incoming international travel volume via I-94 arrival forms. Additionally, the Office of Immigration Statistics within DHS reports immigration statistics by country of birth or citizenship. In particular, non-immigrant admissions data estimate the volume and characteristics of foreign travellers entering the USA by month for tourism, business, work, education or cultural exchange programmes. Using these data sources, 176 potential source countries were identified for which detailed international travel records to and from the USA were available (figure 1).

### (b) Pathogen source strength, *S_i_*: distribution and prevalence at points of origin

The quantification of pathogen source strength indicates how likely a traveller from a particular country will act as a carrier or transporter for pathogen introduction. Global pathogen data were primarily collected from peer-reviewed publications, global pest and disease databases, and key international health organization reports (e.g. World Health Organization (WHO), Centers for Disease Control and Prevention (CDC); electronic supplementary material, table S2). Each of the 176 countries were ranked by their respective pathogen status (i.e. epidemic phase, prevalence, distribution, duration (years post-introduction), host population coverage, reported cases) and subsequently standardized for unbiased comparison. To that end, the pathogen source strength is a general function of the available data and pathosystem of interest.

Noting that there are various ways to describe the strength of pathogen source using epidemiological measures for a specified space and defined period of time, we illustrate several general approaches to formulating the source strength function, $S_{i}$. In cases of rapidly emerging outbreaks, refined spatio-temporal quantification may be possible. During the 2015–2016 Ebola virus epidemic in West Africa, the CDC reported weekly to monthly Ebola case counts for outbreak countries [19]. With incremental situation reports, the spatio-temporal dynamics of pathogen spread within source countries can be incorporated into the structure instead of assuming pathogen stationarity. For example, we consider the log transformation (to balance weighting for heavily skewed data) of the reported Ebola cases ($C_{i}$) to determine the pathogen strength over time as the epidemic evolves in West African source countries (figure 4*c*)

2.1 $$S_{i}(t)=\log\;(C_{i}(t)+1).$$

On the other hand, for a plant pathosystem, factors such as the prevalence ($P_{i}$), host distribution ($H_{i}$) and infection duration ($D_{i}$) in source countries can be considered. The influence of disease status in the source country, whether directly or indirectly through host distribution, can be represented by an appropriate expression. It is important to note that different expression formats can be used to accurately represent pathogen source strength. For example, plum pox virus (PPV) has existed in Europe for multiple years with widespread prevalence, and therefore, the pathogen source strength can estimate the relative disease situation in each source country by

2.2 $$S_{i}(t)=P_{i}(t)D_{i}(t)\log\;(H_{i}(t)+1).$$

Assessment of the pathogen strength plays an inherent and influential role in the pathway model. Reporting of incursions is often incomplete or lags behind actual spread, given the dynamic nature of pathogen dispersal. Therefore, if a country reports the presence of a pathogen, there is no guarantee that areas relatively nearby are free of infection or infestation. Unreported cases in non-source countries result in underestimates of source strength and commensurate underestimates of pathogen introduction risk [20]. It is important to include all infected countries to avoid underestimation of risk. For example, during the 2016–2017 Zika epidemic, the virus spread quickly through the Americas, but due to the magnitude of infected cases and asymptomatic proportion, confirmed case reporting was severely delayed. Taking into consideration infection uncertainty in adjacent (suspected source) countries can mitigate these underestimations. We provide two methods to capture this additional potential risk of unreported cases: (i) assign a probability of infection to each suspected country via expert opinion and suitability for pathogen presence or (ii) assume that all countries within a certain proximity from a known source country border (e.g. 0 – sharing land boundary, 10, 100, 1000 km) are non-reporting and assign appropriate source strength to each by expert opinion and suitability. The census-travel model can then investigate introduction scenarios accounting for estimated disease prevalence in unknown source countries.

### (c) Connectivity: linkage between source and endpoint

The spatially specific risk for travel-related pathogen introduction varies depending on traveller characteristics and their final destination. Although international passengers are required to declare travel destinations upon entry by the US Customs and Border Protection, detailed itineraries are not readily available due to data sensitivity and complexity. Therefore, in order to estimate the final destination travel pattern at refined spatial scales, we use foreign-born population demographics collected through the US Census and American Community Survey (ACS) databases. Foreign-born refers to anyone who is not a US citizen at birth, including naturalized citizens, legal permanent residents and temporary migrants. As an ongoing survey by the US Census Bureau, the ACS provides annual information on demographics and socioeconomic factors for the entire US population at the census tract level. Census tracts are semi-permanent portions of counties with populations of 1200–8000. Rural census tracts cover larger land areas than dense urban tracts. Although census tracts are a convenient spatial hierarchy to parse population data, the US Census Bureau also provides summarized demographic and economic data at other spatial scales, e.g. county, subcounty, metropolitan area, school district and zip code. We extracted 5-year estimates for foreign-born populations by country of origin/birth, age and sex. Assuming strong social and cultural connectivity (visiting family and friends) is a major driver of human travel, we can then distribute the international traveller volume from each source country across the endpoint region at the selected spatial scale.

### (d) Susceptible host population at endpoint

International travel presents opportunities for introduction across the globe; however, after an initial introduction, a susceptible host population and suitable conditions are necessary for establishment and subsequent spread. For human pathogens such as Ebola (figure 4), Dengue (electronic supplementary material, figure S4), Chikungunya (electronic supplementary material, figure S5), Zika (electronic supplementary material, figure S6) or Chagas (electronic supplementary material, figure S7), we can use human population density (and vector population distributions when applicable) at the selected scale directly. We can also include socioeconomic factors to further refine the vulnerability to spread after an introduction. In particular, impoverished areas have been heavily linked to disease spread [21]. On the other hand, for animal and plant pathogen incursions, the susceptible population needs to be estimated as these distributions are not necessarily widely available and complete. For commercial crops and livestock, we use geospatial maps of commercial farms (often collected by the agricultural commodity, Animal and Plant Health Inspection Service (APHIS), etc.), and parse the spatially distributed population at the same desired scale, connecting the host to the travelling human population from international source(s). In some cases, the susceptible host populations for plants and animal pathogens/biota can be further refined. For example, in areas where horticultural crops are grown, such as *Citrus* and *Prunus*, homeowners often also grow such plants in their dooryards and gardens. In fact, data from past surveys of citrus diseases conducted by state/federal regulatory agencies show approximately 60% of residences in Florida grew citrus in their gardens, at approximately two trees per residence. When similarly validated datasets are available, we can link residential host plants to the distribution of human residences at the chosen spatial scale as a proxy to estimate residential host plant populations. We then join the resulting residential and commercial plant host populations to approximate the spatial distribution of hosts within the endpoint area of interest at fine to coarse granularity.

### (e) Risk algorithm and mapping

The deterministic model is a sum of the accumulated risk components from source countries ($R_{\mathrm{SC}}$), adjacent suspected countries ($R_{\mathrm{AC}}$) and returning outbound US travellers ($R_{\mathrm{OUT}}$) for each parcel *j*. Each risk component is weighted by the user ($W_{\mathrm{SC}},W_{\mathrm{AC}},W_{\mathrm{OUT}}$) to calculate the total risk for pathogen introduction through the appropriate travel pathways, $R_{T}$ (equations (2.3–2.6)). For each land parcel *j* within the regional landscape made up of land parcels {1,2,⋯,M}, the parameter $F_{ij}$ denotes the foreign-born population of country *i* to estimate the likelihood of external disease introduction through interaction (e.g. contacts, activities), whereas $P_{j}$ denotes US population. The inbound and outbound travel volume of the interested demographic group (e.g. passenger visa class, age, sex, travel month) to/from country *i* are estimated as $T_{i}\;$ and $O_{i}$, where *N* and *K* are the number of pathogen sources and adjacent suspected countries, respectively. The nearest distance between country boundaries is calculated as $d_{ik}$, where the user-defined maximum distance threshold value $d_{T}$ filters the areas included in the adjacent country risk.

2.3 $$R_{\mathrm{SC},j}=\overset{N}{\underset{i=1}{\sum }}\left(\frac{F_{ij}}{\sum_{\;j=1}^{M}F_{ij}}\right)T_{i}S_{i},$$

2.4 $$R_{\mathrm{AC},j}=\overset{K}{\underset{k=1}{\sum }}\left(\frac{F_{kj}}{\sum_{\;j=1}^{M}F_{kj}}\right)T_{k}S_{k},\;\mathrm{if}\;d_{ik}\leq d_{T},$$

2.5 $$R_{\mathrm{OUT},j}=\overset{L}{\underset{l=1}{\sum }}\left(\frac{P_{j}}{\sum_{\;j=1}^{M}P_{j}}\right)O_{l}S_{l},\;\mathrm{where}\;\;L=N+K,$$

2.6 $$R_{T,j}=W_{\mathrm{SC}}R_{\mathrm{SC},j}+W_{\mathrm{AC}}R_{\mathrm{AC},j}+\;W_{\mathrm{OUT}}R_{\mathrm{OUT},j}$$

2.7 $$\mathrm{and}\;\mathrm{CR}I_{z,j}=R_{T_{z},j}/\underset{l\in \{1,\ldots ,L\}}{\max}(R_{T_{l},j}),\;\mathrm{where}\;z\in \{1,\ldots ,L\}.$$

**Figure 2.**
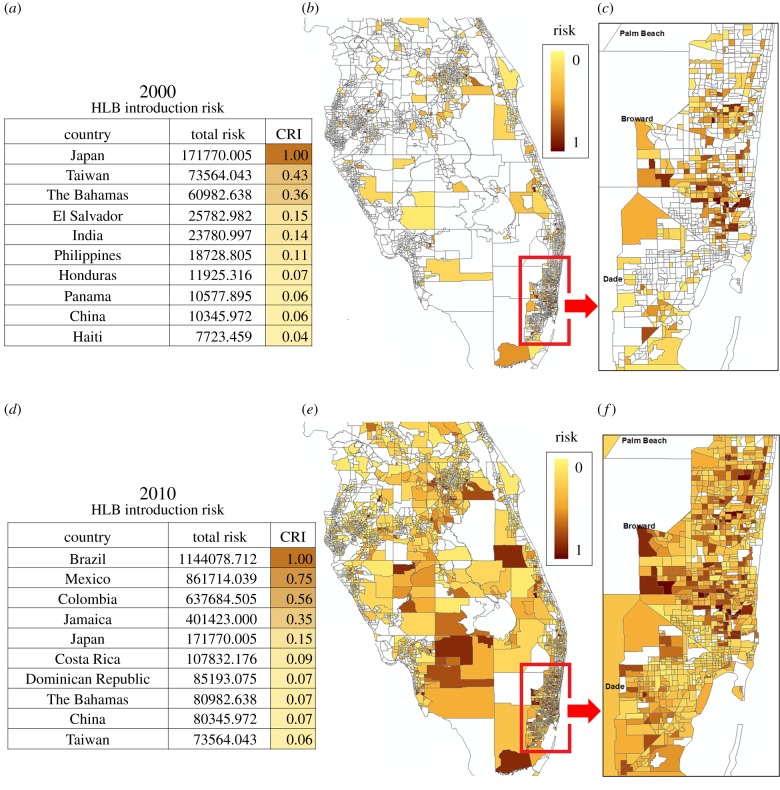
Census-travel model introduction risk for citrus HLB in Central and South Florida citrus-producing regions, comparing retrospective and recent risk estimates (note that HLB spread within/between USA is not explicitly considered). (*a,d*) Source CRIs for the top 10 countries of highest risk contribution in 2000 and 2010, respectively. Note, over time, Brazil emerged as the largest contributor of HLB risk of introduction for Florida. (*b,e*) Introduction risk estimates by census tract for 2000 and 2010. (*c,f*) Higher resolution metropolitan Miami demonstrating the shift in risk distribution for introduction points.

For visualization of the risk distribution, we link the output file with a geographical information system (GIS) mapping program such as ArcGIS© to generate risk maps. Risk estimates are updated as various factors are changed and explored by the user and new scenarios are calculated. Each parcel is assigned an overall risk index, and the population of parcels are segregated into user-defined risk categories (incremental steps of risk). For convenience, we normalize risk estimates on a 0.0–1.0 scale (figure 2). From a regulatory standpoint, surveys are often deployed inclusive of regional political boundaries, such as state, province, county, etc. Thus, risk maps are scalable such that the users can interrogate the entire region (figure 2*b,e*) or subregions at higher resolution (figure 2*c,f*). The risk contribution by various countries can be ranked via a ‘Country risk index’ (CRI), where $R_{T,i}$ is the total risk from country *i* (equation (2.7)). Because the CRI integrates the disease situation and international travel volume, its range of values is broad and therefore normalized to a 0.0–1.0 scale for better comparison (figure 2*a,d*). It is important to note that a retrospective analysis of the census-travel model can be powerful in determining the historical introduction dynamics and predicting near-term future introduction patterns.

## 3. Results

### (a) Multiple pest/disease survey and deployment

**Figure 3.**
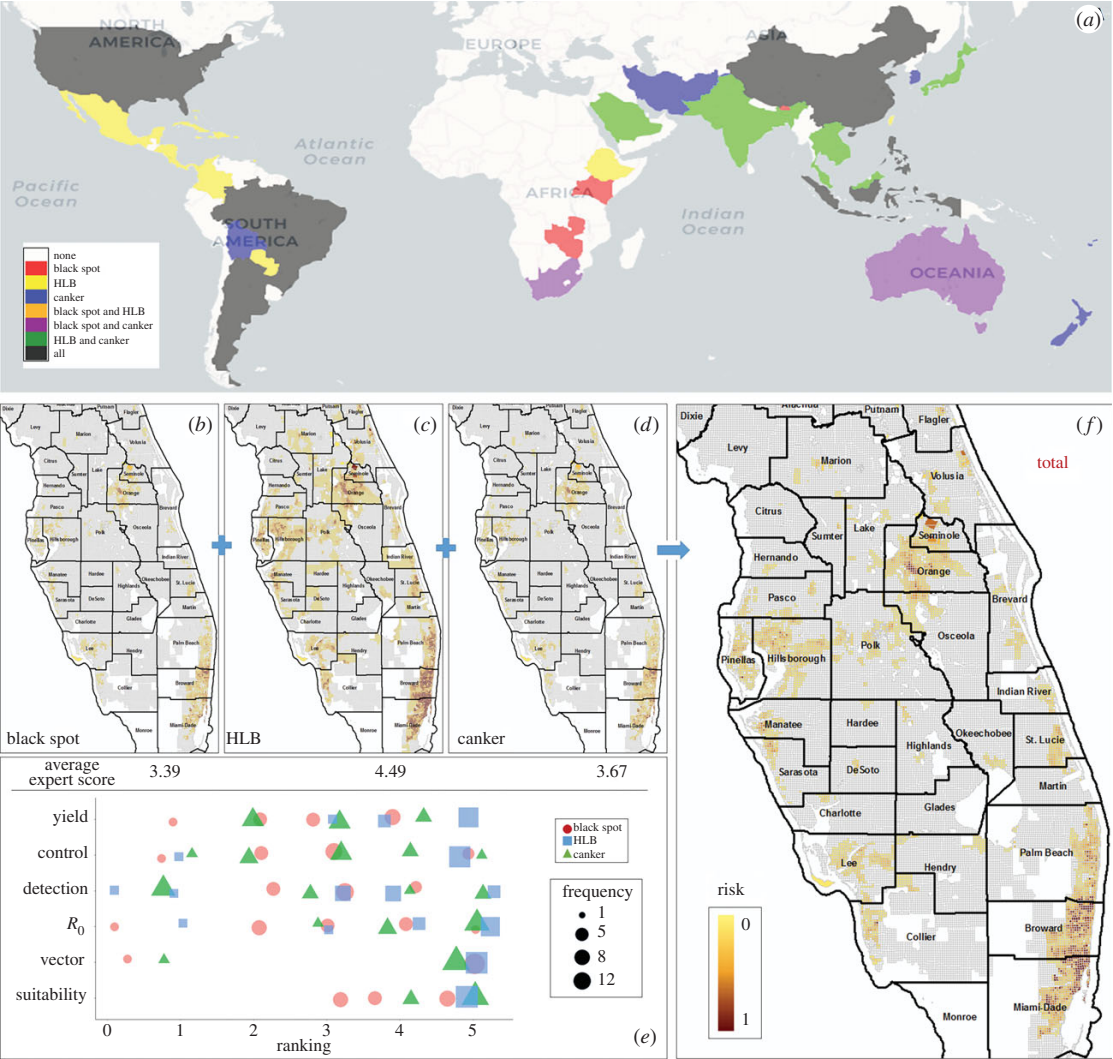
Procedure for multiple pathogen/disease survey using census-travel model risk output. (*a*) Global disease distribution for citrus black spot, citrus HLB and citrus canker. (*b–d*) Individual risk maps for Florida introduction in 2010. (*e*) Expert opinion scores on suitability for spread, vector prevalence (if applicable), reproduction rate (*R*_0_), detection and confirmation inefficiency, control cost(s) and yield reduction/crop damage for each disease (score ranking: 1, low; 5, high). (*f*) Combined overall risk map used for survey prioritization. For visualization, risks are summarized at 1 mi^2^ to meet US regulatory agency survey implementation requirements.

Surveys are costly in manpower and fiscal resources. Therefore, it can be advantageous to survey for multiple exotic pathogens/pests simultaneously. For example, we have developed a multiple exotic citrus pathogen surveillance programme that routinely includes Asiatic citrus canker, citrus Huanglongbing (HLB) and citrus black spot (among many others) that has been deployed by the USDA, APHIS and State of Florida for several years [22]. Via this survey programme, both emerging pathogens and those yet to be introduced or discovered are integrated into a single comprehensive survey. The census-travel model allows the simultaneous inclusion of multiple pathogens and provides the methodology to rank the pathogens by their relative importance. This user-defined ranking can be based on perceived pathogen impact/concern on the commodity if introduced, e.g. environmental/climatic suitability for disease development, prevalence of associated vector, reproduction rate, detection/confirmation limitations, cost of control/eradication if introduced, estimated crop damage on yield/quality or by socio-political importance. To make sound science-based decisions on disease priority prior to survey implementation, we adopt procedures to elicit structured expert opinion on disease ranking. For example, figure 3 illustrates the multi-pathogen survey approach using the census-travel model. Twelve anonymous independent experts ranked six epidemiological factors for citrus canker, black spot and HLB in Florida in 2010. The census-travel model calculates the risk of introduction for each pathogen separately (figure 3*b–d*), before weighting them according to their ranking (figure 3*e*) to generate an aggregate risk output for each land parcel. The overall risk by parcel can then be used to partition survey efforts across the region (figure 3*f*).

For either individual pathogen or multi-pathogen surveillance, regional survey (e.g. state-wide) requirements are incorporated in a spreadsheet table that uses the overall prioritized risk categories of introduction to calculate the number of sampling points within each designated land parcel. This table can be used as a ‘scenario generator’ to estimate fiscal, personnel (surveyors, supervisors, support staff, etc.), vehicle, equipment and miscellaneous requirements needed to survey for new introductions. By changing the sampling frequency, i.e. the number of samples assigned to parcels by their risk category, the overall logistical requirements of the survey are conveniently recalculated and can be used by regulatory agencies to explore the possibilities of apportioning manpower and fiscal resources in various ways to achieve regulatory goals.

When developing targeted survey methodologies, it is important to not bias efforts exclusively towards areas with higher risk because of the uncertainty that all risk factors influencing introduction and propagation are known and taken into account. Therefore, the census-travel model output of new/continuous introduction risk can be readily integrated with additional risk factors capturing risk from prior introductions, post-introduction spread and vector population distributions, among a variety of other epidemiological or social risks. In fact, the model has already been incorporated into various federal and state regulatory risk-based surveillance programmes as a major component of risk. Two recent deployments of the census-travel model include its incorporation into PPV surveys for New York since 2013, and more recently, for early detection in California, where introduction risk output is combined with other risk layers to generate an overall risk map (electronic supplementary material, figure S1A,B). Similarly, the census-travel model generates a risk component for continual introduction of *Candidatus* Liberibacter asiaticus (*CLas*) which has been incorporated into ongoing *CLas*/ACP surveys in California [23,24] (electronic supplementary material, figure S2). In both cases, the construction of the supplementary risk components is beyond the subject of this paper.

### (b) Model application demonstration and validation

**Figure 4.**
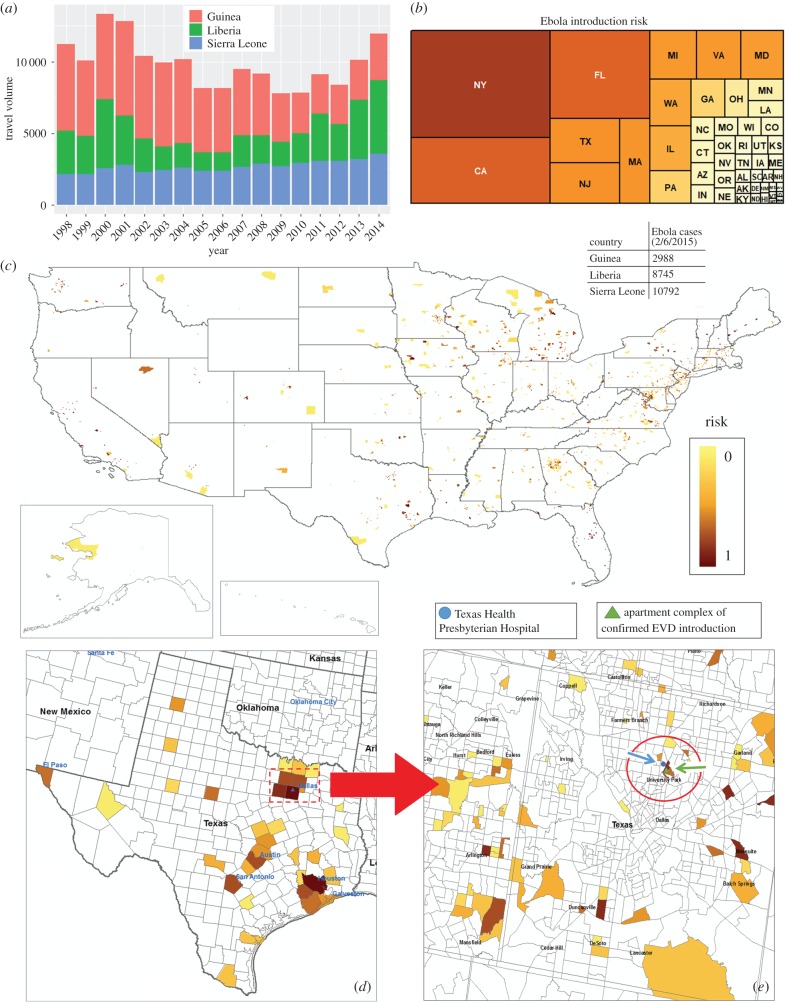
(*a*) Annual travel volume by year into the USA from West African countries, and (*b*) aggregated Ebola risk distribution by state (indicated by state abbreviation). Census-travel model output for 2015 Ebola (EVD) risk introduction predictions for the entire USA (*c*), Texas (*d*) and the Dallas metropolitan area (*e*) by land parcel (census tract) using the log transformation of number of cases from infected West African countries. Actual introduction site (apartment complex; green triangle and arrow) and attending hospital (blue circle and arrow).

The census-travel model has predicted introduction risk for many pathosystems across the USA (electronic supplementary material, table S1). A compelling validation of the predictive power of the census-travel model was the prediction of Ebola introduction into the USA in 2015. Using reported Ebola case numbers from infected West African countries (Guinea, Liberia, Sierra Leone), the census-travel model provided risk estimates for Ebola virus introduction in parcels (census tracts) for the entire USA, highlighting Texas, and metropolitan Dallas regions (figure 4*c–e*). The model captures the general travel pattern from the source countries, predicting several high-risk areas across the USA including the precise census tract where an introduction occurred. A Liberian man inbound from Africa had developed symptoms after arrival into the USA and sought medical attention at the nearest hospital to his residence (location of apartment complex and hospital indicated, figure 4*e*). Linking medical facilities with areas at high risk for introductions can assist in prioritizing training and detection services for new emerging diseases. Figure 4 also illustrates the flexibility of the census-travel model in predicting disease introduction risk at various spatial scales, i.e. state, county, census tract, TRS (township-range-section).

Additional model validation for plant pathosystems at the TRS level have been included in electronic supplementary figures S1 and S2, highlighting the prediction accuracy and reliability of the census-travel model. For example, the census-travel model was used to predict *CLas* introduction into California in 2010. HLB was initially detected in Southern California in 2012 (2 finds) and again in 2015 (15 finds). The census-travel model estimated the introduction risk to be greater than 0.65 (on a standardized 0–1 scale) in approximately 4.6% of the TRSs covering Southern California. Assuming that 300 TRSs (i.e. 5% of the Southern California TRSs) are selected completely at random for HLB survey, the probability that all TRSs from the initial 2012 and 2015 HLB+ detections are included is 0.00000536 (*p* < 0.001), according to binomial probability theory. On the other hand, selecting the top 5% highest risk estimates from the census-travel model for survey, all detected HLB+ TRSs will be covered.

### (c) Online model interface

An interactive graphical user interface (https://epi-models.shinyapps.io/Census_Travel/) was created in Shiny (web application framework for R) to allow users to parametrize the model via multiple input screens, execute it and inspect graphical outputs of source and endpoint connections and risk maps for introductions into the USA (electronic supplementary material, figure S3). We have compiled a list of global pathogen/disease datasets (electronic supplementary material, table S1) for online model demonstration and testing. The user can select various pathogen(s)/disease(s) to investigate or upload their own global pathogen data. For each pathogen of interest, the user can define the relative weightings for a multi-pathogen survey approach. Additionally, components such as weighting for source/adjacent/outbound travel, census year for socioeconomic and demographic input data (i.e. retrospective versus current introduction risk analyses), US endpoint state(s), land parcel type (spatial risk output level) and traveller details (age, gender, etc.) are available to the user to run the census-travel model under different scenarios. Possible introduction pathways between source countries and endpoint areas can be further investigated using the integrated three-dimensional interactive map via a Google Earth interface. This interface allows the user to visually explore which country has the largest contribution of risk, as well as relative risk contributions by country, indicated by bar heights extending away from each origin country (electronic supplementary material, figure S3B). The user can also select the granularity of the on-screen maps to visualize initial output. The selected visual granularity does not affect the output file which contains all output data requested. The output files are available for download, and can be linked to a GIS mapping programme to display maps at user-identified spatial scales. The same output files can be used to develop surveillance programmes using the risk ranking to apportion resources (i.e. the number of surveyors, samples to be taken) within available manpower and fiscal constraints. An informative documentation file is also provided to assist users with running the model.

## 4. Conclusion

The census-travel model allows the user to explore potential points of introduction of a wide range of exotic biota, including contagions such as plant, human and animal disease pathogens. To our knowledge, this is the first demonstration that integrates global travel data to predict/propagate pathogen introduction risk at fine spatial scales for efficient survey design and implementation (e.g. census tract or TRS), whereas all other methods calculate risk at coarser county or state scales. This modelling framework is highly flexible, and can be similarly employed to estimate introductions in other countries and various endpoint scales (i.e. land parcel size) when detailed travel and census databases become available. The versatile model has a multitude of direct applications and potential for extensions.

We have initially focused on international travel as the primary driver of pathogen introduction, i.e. primary spread. Possible extensions to the census-travel model include incorporating international trade. However, there is a significant lack of publicly available, comprehensive trade data on the global scale currently, which adds tremendous difficulties on conducting rigorous international trade pathway analysis. After arrival at a particular port-of-entry, data on the distribution towards secondary and tertiary points for dissemination are typically proprietary, if it exists at all, and thus, the risk output spatial scale would require aggregation to large scales (i.e. county or state level), losing the granularity of the census-travel model for efficient survey design and implementation.

The census-travel risk map highlights the potential areas for introduction to optimize resource allocation for a surveillance programme. However, the chance of introduction is still a stochastic process and international travel may not be the only contributing factor for pathogen introduction. Furthermore, identification of detected infections resulting from primary versus secondary spread is not always clear, due to lack of reporting and potential sampling bias. Depending on the pathosystem, particularly after initial introductions, other risk factors should be taken into account to prioritize survey programmes to consider secondary spread mechanisms. To that end, we have incorporated the census-travel model as a major risk component of other risk-based models that are used to drive pathogen surveys. For example, we have incorporated census-travel risk in the PPV risk-based survey model, generated annually since 2013 for New York State, which serves as the basis of the PPV eradication and early detection survey in New York and California. Census-travel risk has also been incorporated into the ACP/HLB risk-based survey models for California, Florida and Texas. Resulting surveys over the past 7 years have led to the discovery of greater than 1200 detections of *CLas*-infected trees in California's Los Angeles Basin (electronic supplementary material, figure S2). Many of the high-risk introduction TRSs capture the HLB finds in the early years, although the predictability by the census-travel model alone decreases gradually after HLB establishment and subsequent secondary spread increases. The model has also served as the basis of the Florida risk-based survey for multiple citrus pathogens conducted yearly by a joint state–federal Cooperative Agricultural Pest Survey (CAPS).

Additionally, the model output can be used to inform epidemiological calculations. After the contagion is found, pathogen incidence can be estimated based on survey intensity metrics [25,26]. Furthermore, due to the probabilistic nature of the model, it can be used as the spatio-temporal starting point for modelling the potential spread of pathogens via epidemiological models or estimating the contact ratio within an existing area. For example, by integrating susceptible host population densities in an area of concern (data source: US census) and social interactions (distances to schools, shopping, restaurants and radii of probable work travel distances, etc.), we can estimate the contact ratio. Contact ratio can be combined with duration of infectiousness and *R*_0_ of the contagion to predict spatial and temporal spread into surrounding areas. Such estimations can also serve as a driver for responding to potential outbreaks. Response can involve logistical planning such as staging manpower and resources in strategic locations of high risk and potential spread as an early response to control or mitigate the pathogen.

## Supplementary Material

Data, model structure and applications

## Data accessibility

This article has no additional data.
