# Supplementary material for: A probabilistic census-travel model to predict introduction sites of exotic plant, animal and human pathogens
Source: Philos Trans R Soc Lond B Biol Sci. 2019 May 20;374(1776):20180260. doi: 10.1098/rstb.2018.0260 (PMC6558561; doi:10.1098/rstb.2018.0260)
Supplement: Data, model structure and applications [file rstb20180260supp1.docx]

**Supplementary Materials**

Table S1. Examples of phytobacterial, phytoviral, phytomycological, and zoonotic viral vectored and non-vectored pathogens, where the census-travel model has been applied to predict new and recurring introduction points. A representative subset has been illustrated in the manuscript and supplement highlighting the utility and versatility of the model.

| **Pathosystems modeled** | Pathogen | Possible Vector | Example Output | Example Parcel Size |
| --- | --- | --- | --- | --- |
| Zoonotic disease |  |  |  |  |
| Ebola | Ebola virus (EV), Ebolavirus | Human contact | *Fig 4c-e* | *State*  *County Census tract* |
| Zika | Zika virus (ZIKV), Flavivirus | *Aedes aegypti* and *A. albopictus*, human contact | *Fig S6* | *County Census tract* |
| Dengue | Dengue virus (DENV), Flavivirus | *Aedes aegypti* | *Fig S4* | *Census tract* |
| Chikungunya | Chikungunya virus (CHIKV), Arbovirus | *Aedes aegypti* | *Fig S5* | *Census tract* |
| Yellow Fever | Yellow fever virus (YFV), Flavivirus | *Aedes aegypti* |  |  |
| Malaria | Protozoa Plasmodium falciparum | Anopheles *mosquito* |  |  |
| Chagas disease (syn American trypanosomiasis) | *Trypanosoma cruzi* | Triatomine insects, exp. *Reduviidae sp., Rhodnius prolixus, (Kissing bug) etc.* | *Fig S7* | *Census tract* |
| Rift Valley Fever | Rift valley fever virus (RVF), Phlebovirus | *Aedes aegypti* |  |  |
| Phytobacterial |  |  |  |  |
| Huanglongbing (HLB) | *Liberibacter asiaticus* (Las) | *Diaphorina citri* | *Fig 2, Fig 3c, Fig S3* | *Census tract*  *TRS grid* |
| Citrus greening (CG) | *Liberibacter africanus* (Laf) | *Triosa eryatrae* | *Fig S3* | *Census tract* |
| American citrus greening (ACG) | *Liberibacter americanus* (Lam) | *Diaphorina citri* | *Fig S3* | *Census tract* |
| Asiatic citrus canker (Acc) | *Xanthomonas citri* pv *citri* | Rain splash, contact | *Fig 3d* | *TRS grid* |
| Citrus Variegated Chlorosis (CVC) | Xylella fastidiosa | Sharpshooters |  |  |
| Phytoviral |  |  |  |  |
| Citrus leprosis | Citrus leprosis virus (CiLV-N, CiLV-C), Dichorhavirus | *Brevipalpus sp*. (phytophagus mite) |  |  |
| Plum pox (syn. Sharka) | Plum Pox Virus (PPV), Potyvirus | Multi aphid species incl,: *Myzus persicae, Brachycaudus cardui, Brachycaudus helichrysi,* etc. | *Fig S1a* | *TRS grid* |
| Phytomycological |  |  |  |  |
| Citrus black spot (CBS) | *Phylosticta citricarpa, (*syn*. Guignardia citricarpa*) | Airborne ascospores and Rain splash, mechanical | *Fig 3b* | *TRS grid* |
| Sweet orange scab (SOS) | *Elsinoë australis* | Rain splash | *Fig S8* | *Census tract* |
| Gladiolas rust | *Uromyces transversalis* | Aerial – wind blown |  |  |

| Model component | Source | Availability | Access | Data Description |
| --- | --- | --- | --- | --- |
| International Travel | Office of Immigration Statistics | 2000 - present | <https://www.dhs.gov/immigration-statistics/yearbook> | Nonimmigrant travel admissions by visa class, country of citizenship, age, sex and month of arrival |
|  | National Travel and Tourism Office | 1995 - present | <http://travel.trade.gov/> | Auxiliary information about US inbound and outbound travel and tourism statistics |
| Pathogen Source | Global Peat and Disease Database  GPDD | Current | <https://www.gpdd.info/> | Pest information repository pertaining to pest taxonomy, identification, biology, distribution, hosts, significance, detection and control with over 6000 pests either not known to be in the US or present, but with a limited distribution. |
|  | Global Burden of Disease | 1990 - 2016 | <https://vizhub.healthdata.org/gbd-compare/> | Comprehensive study tracking and summarizing more than 400 diseases, injuries and risk factors in the US |
|  | Scientific literature review;  Expert elicitation | Current |  | Temporal, spatial and biological characteristics of outbreaks, epidemiological profiles of disease by country (cases, mortality, infection areas, etc.) |
| Connectivity | American Community Survey | 2006 - present | <http://www.census.gov/acs> | Annual information on demographics and socioeconomic factors for entire US (foreign-born population by birth country, age, sex, etc.) |
|  | TIGER/Line® Shapefiles | 2000 & 2010 | <ftp://ftp2.census.gov/geo/tiger/TIGER2010/> | Spatial format used by the US Census Bureau to describe land attributes and features such as county, census tracts, zip code, etc. |

Table S2. Data sources mined for census-travel model development.


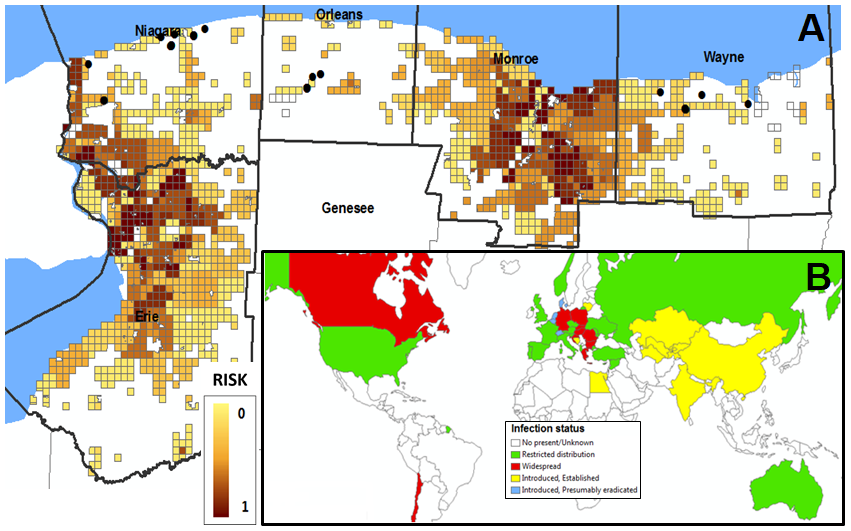


Figure S1. Deployments of the census-travel model include its incorporation into two surveillance protocols in New York and California. The risk outputs have been parsed into 1-mi^2^ parcels (individual squares) for both. (A) Plum pox virus (PPV) introduction risk map included into New York’s risk-based survey protocol. Positive PPV detections at commercial prunus orchards are displayed as black dots. (B) Global PPV infection status used to construct pathogen source strengths.


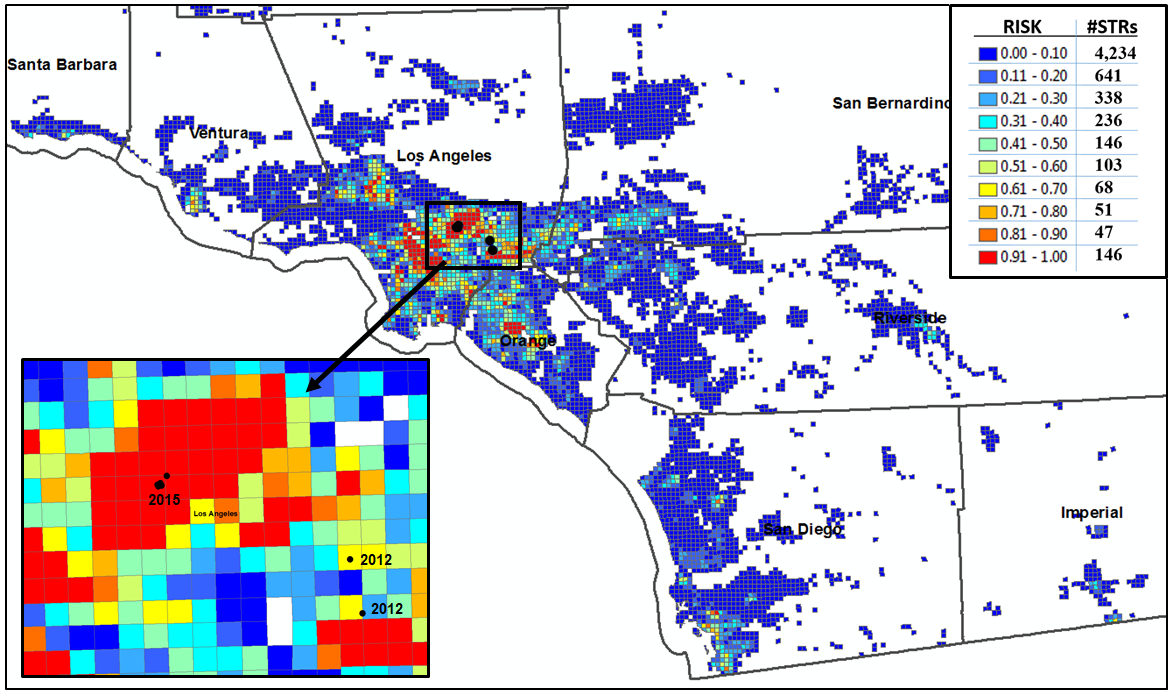
Figure S2. Census-travel model risk prediction for *CLas* introduction in California in 2010. Initial confirmed HLB+ locations from Tree and ACP samples in 2012 and 2015 are indicated by the black dots (2 sites in 2012, 15 sites in 2015). No new HLB+ detections were found in 2013-2014. The inset zoom-in map surrounding the earliest detections illustrates the performance of the census-travel model prediction at TRS level. The model predicts reasonably well for HLB+ finds (TRS Risk = 0.65) in 2012, and extremely well for all finds (TRS Risk = 1.0) in 2015.


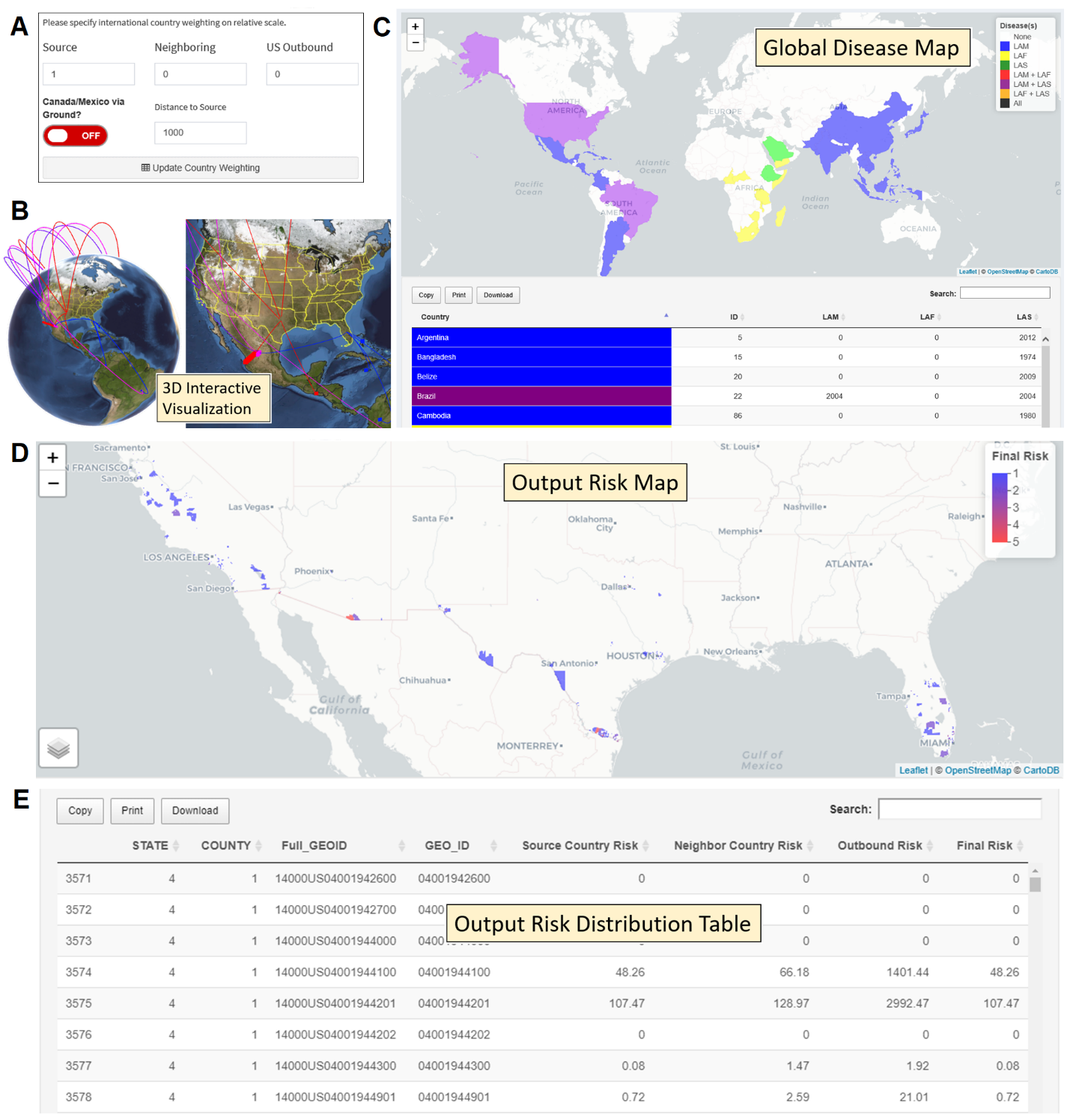


Figure S3. Screenshots of the census-travel model web application. (A) The interface provides numerous user-defined inputs including endpoint state(s), pathogen source strengths, weightings for source, adjacent and outbound travel, rankings for a multi-pathogen survey design, and the output land parcel spatial scale. After selecting or uploading global pathogen data, the interface provides as an interactive map (B) as well as a global disease map (C) to assist the user with potential introduction pathway visualization. Once the user runs the model, risk outputs are generated at the desired scale. (D) An example risk map output at the census tract level for introduction risk into Arizona, California, Florida and Texas, and the corresponding risk distribution table output file (E). The generated risk outputs are available to download.

Application link: <https://epi-models.shinyapps.io/Census_Travel/>


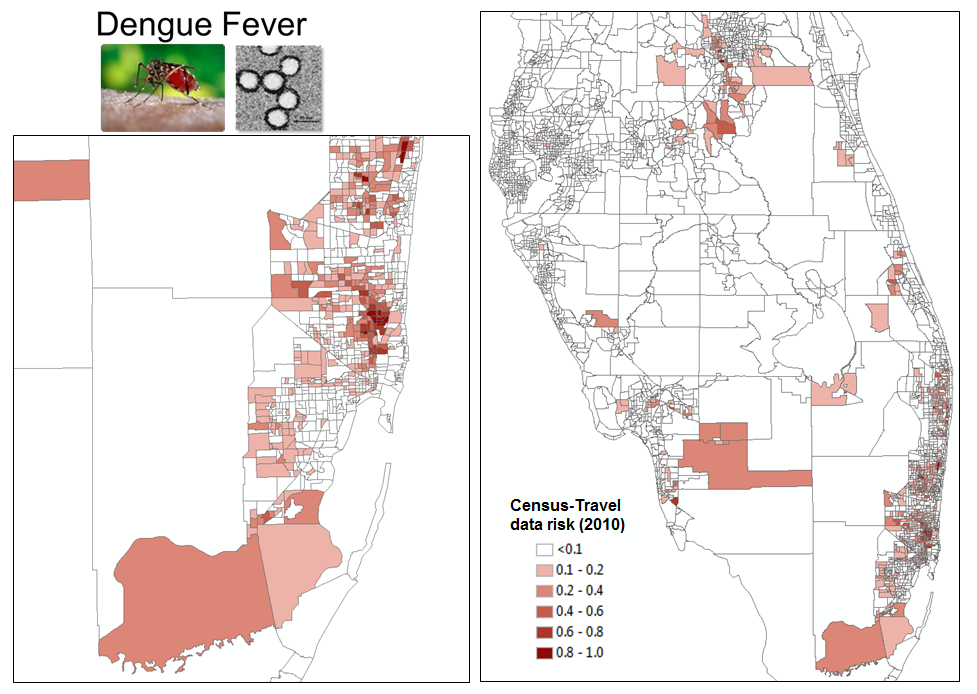


Figure S4. Census-travel model estimates of risk of introduction of Dengue Fever Virus into Florida and Miami area, USA.


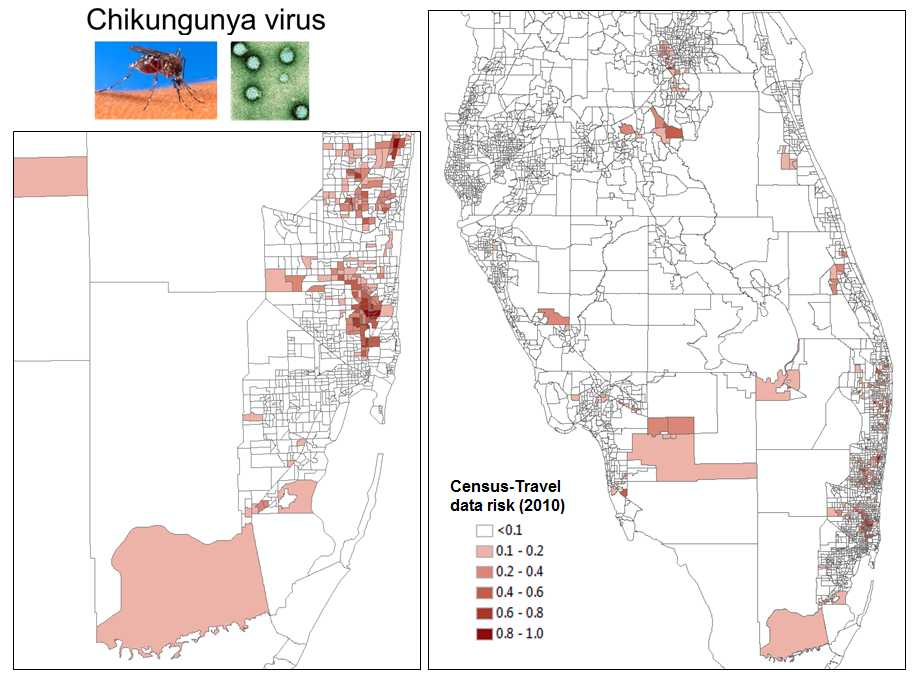


Figure S5. Census-travel model estimates of risk of introduction of Chikungunya Virus into Florida and Miami area, USA.


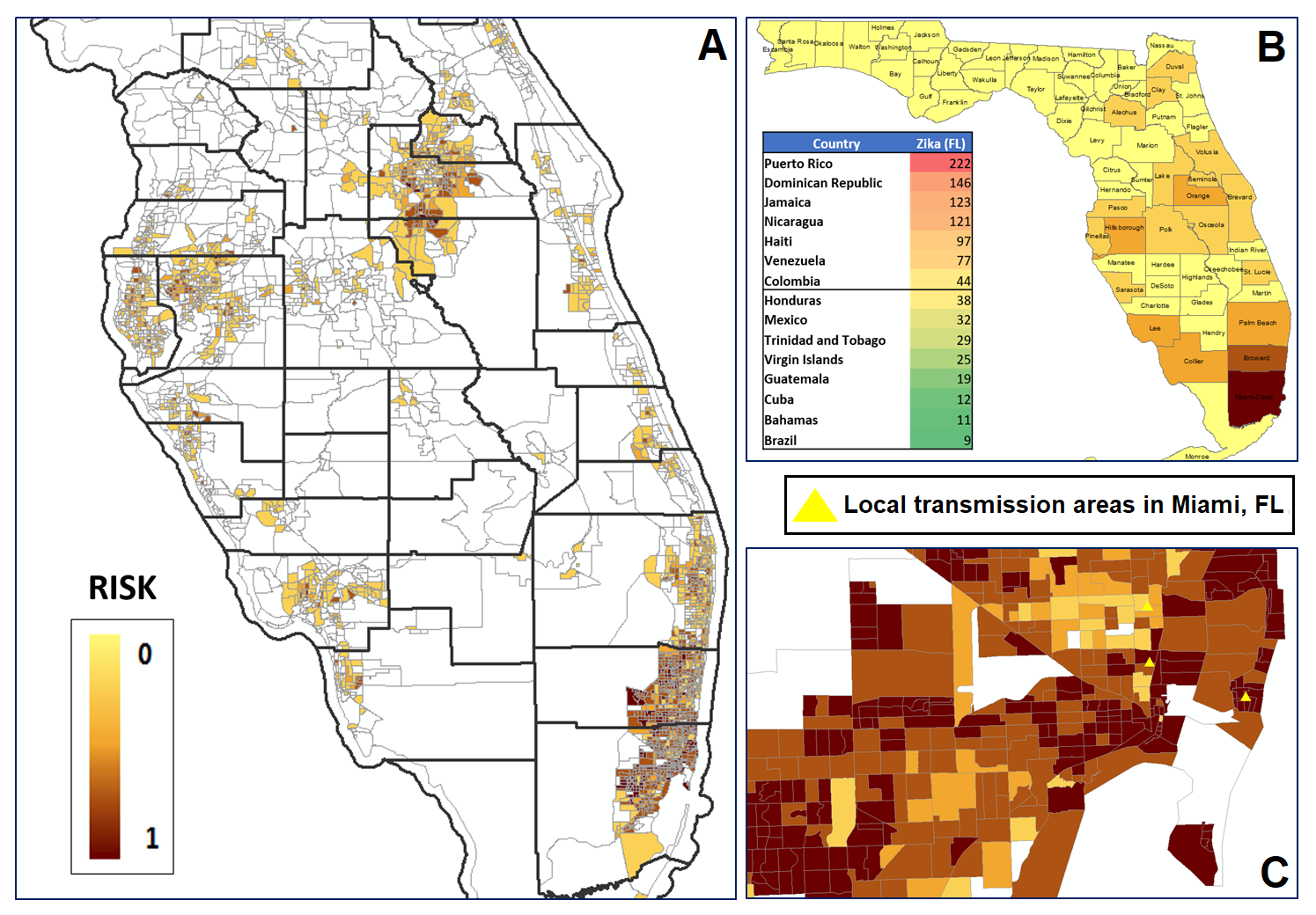
Figure S6. Census-travel model estimates of risk of introduction of Zika Virus into Florida and Miami area, USA in 2016.


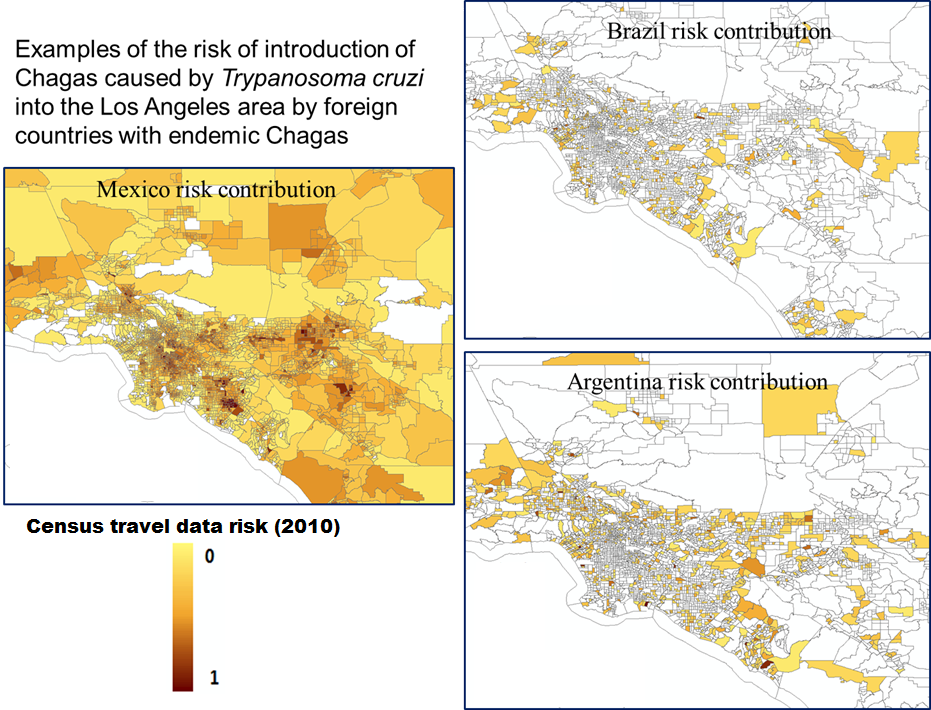


Figure S7. Census-travel model estimates of risk of introduction of Chagas into Los Angeles Basin of California, USA. Figure demonstrates relative risk contributions by various potential source countries.


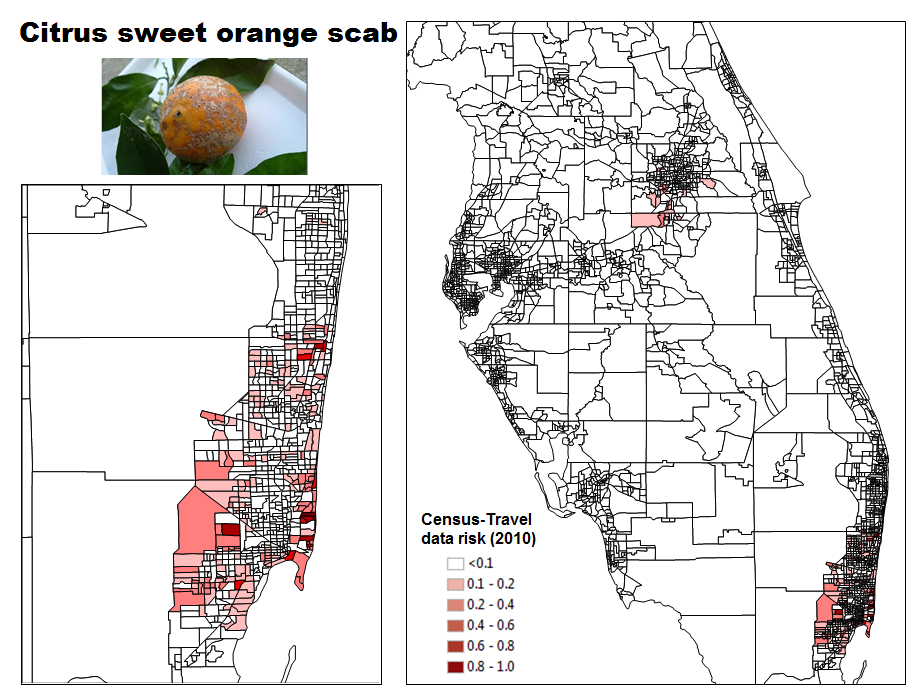


Figures S8. Census-travel model estimates of risk of introduction of citrus sweet orange scab into Florida and Miami area, USA.
